# Coxsackievirus A16 Elicits Incomplete Autophagy Involving the mTOR and ERK Pathways

**DOI:** 10.1371/journal.pone.0122109

**Published:** 2015-04-08

**Authors:** Yingying Shi, Xiaohua He, Guoguo Zhu, Huilin Tu, Zhongchun Liu, Wenhua Li, Song Han, Jun Yin, Biwen Peng, Wanhong Liu

**Affiliations:** 1 Pathogenic Organism and Infectious Diseases Research Institute, School of Basic Medical Sciences, Wuhan University, Wuhan, 430071, China; 2 Hubei Province Key Laboratory of Allergy and Immunology, Wuhan, 430071, China; 3 Hubei Provincial Key Laboratory of Developmentally Originated Disease, School of Basic Medical Sciences, Wuhan University, Wuhan, China; 4 Intensive Care Unit of Emergency Department, Wuhan General Hospital of Guangzhou Command, Wuhan, 430071, China; 5 Institute of Neuropsychiatry, Renmin Hospital, Wuhan University, Wuhan, 430060, China; 6 College of Life Sciences, Wuhan University, Wuhan, 430072, P R China; Saint Louis University, UNITED STATES

## Abstract

Autophagy is an important homeostatic process for the degradation of cytosolic proteins and organelles and has been reported to play an important role in cellular responses to pathogens and virus replication. However, the role of autophagy in Coxsackievirus A16 (CA16) infection and pathogenesis remains unknown. Here, we demonstrated that CA16 infection enhanced autophagosome formation, resulting in increased extracellular virus production. Moreover, expression of CA16 nonstructural proteins 2C and 3C was sufficient to trigger autophagosome accumulation by blocking the fusion of autophagosomes with lysosomes. Interestingly, we found that Immunity-related GTPase family M (IRGM) was crucial for the activation of CA16 infection-induced autophagy; in turn, reducing IRGM expression suppressed autophagy. Expression of viral protein 2C enhanced IRGM promoter activation, thereby increasing IRGM expression and inducing autophagy. CA16 infection inhibited Akt/mTOR signaling and activated extracellular signal-regulated kinase (ERK) signaling, both of which are necessary for autophagy induction. In summary, CA16 can use autophagy to enhance its own replication. These results raise the possibility of targeting the autophagic pathway for the treatment of hand, foot, and mouth disease (HFMD).

## Introduction

Coxsackievirus A16 (CA16) is a positive-strand non-enveloped RNA virus that belongs to the genus *Enterovirus* in the family Picornaviridae[1]. The genome of CA16 is about 7.4 kb in length, with only one open reading frame (ORF) to encode a polyprotein that is composed of four capsid proteins, VP1 to VP4, and seven nonstructural proteins, 2A, 2B, 2C, 3A, 3B, 3C, and 3D[1,2,3]. As VP1 has been verified to contain neutralization antigenic sites and retain evolutionarily conserved, it has been used to track genotypes of CA16-associated hand, foot and mouth disease (HFMD) over different temporal and geographical outbreaks[1,2,3]. CA16 is one of the major causative agents of HFMD, which is characterized by herpetic lesions on the hands, feet and oral mucosa in children less than 5 years old[1,2,3]. CA16 has circulated mainly in the Pacific and Southeast Asia regions in recent decades and has been reported to be responsible for nearly half of all of the confirmed HFMD cases in mainland China, where HFMD has become a serious public health problem[2,3,4,5]. While most CA16-associated HFMD infections present only mild symptoms, many recent reports show that CA16 infections might also lead to severe health issues, such as aseptic meningitis, rhombencephalitis and even death[2,4,5]. Moreover, valid antiviral therapy or a vaccine are not currently available. Therefore, it is important to understand the biology of this virus to develop strategies to control its pathogenicity. In our previous studies, we showed that Coxsackievirus A16 infection triggered apoptosis in rhabdomyosarcoma cells (RD cells) by inducing ER stress[6]. Furthermore, ongoing studies have suggested that both apoptosis and ER stress may be linked to the autophagic response, which plays a vital role in allowing cell survival under stress conditions[7,8]. Therefore, an investigation into the possible involvement of CA16 in the regulation of the autophagic process was a logical next step.

Autophagy is one of the most important homeostatic mechanisms. It involves the formation of double-membrane vesicles called autophagosomes that sequester damaged cytoplasmic organelles, protein aggregates, and invading intracellular pathogens for degradation[9,10]. Autophagy is triggered by various stress stimuli, including nutrient starvation, pathogen-associated molecular patterns (PAMPs) and virus infection [9,10]. More than 30 autophagy-related (ATG) genes have been implicated in this process. For example, Atg5 and Beclin1 play key roles in autophagosome nucleation and elongation, respectively, thereby contributing to autophagosome formation[9,10]. Because autophagosomes are intermediate products of the autophagy process and require fusion with lysosomes to induce degradation, autophagosome accumulation may be the result of either increased autophagosome generation or suppressed degradation[9,10]. Additionally, the mammalian target of rapamycin (mTOR) and the phosphatidylinositol 3-kinase (PI3K) signaling pathways have been reported to be involved in autophagy regulation in mammalian cells[9,10,11]. mTOR is a major negative regulator of autophagy and it receives inputs from different signaling pathways. When mTOR is repressed, ULK1 (Atg1) is released from the mTOR complex, resulting in the hypophosphorylation of ULK1 and Atg13. The activated hypophosphorylated ULK1 then participates in the initial stage of autophagy[9,10,11]. ERK activity also has been reported to be associated with autophagic cell death in response to different stresses, such as amino acid depletion and virus infection[12].

Recently, increasing evidence has demonstrated that autophagy plays a key role in viral infections. Autophagy may function as an intrinsic host defense mechanism via cytoplasmic sampling and delivery of intracellular pathogens or components of these pathogens to TLR-containing endosomes or compartments enriched in major histocompatibility complex (MHC)-II molecules for degradation[13,14,15]. For example, it has been reported that the induction of autophagy upon rapamycin treatment can facilitate the eradication of *Mycobacterium tuberculosis* in macrophages[13]. However, certain viruses have developed different molecular strategies to avoid or exploit this process for their own benefit. For example, herpes simplex virus 1 (HSV-1) has been confirmed to suppress the host cell autophagy response to decrease MHC class I presentation of viral antigens[16]. Conversely, dengue viruses trigger autophagy and utilize amphisomes as a site for translation and replication[17]. However, picornaviruses exhibit inconsistent phenomenon in autophagy induction. For example, coxsackievirus B4 (CVB4) has been confirmed to induce autophagosome accumulation, thereby enhancing virus replication[18]. In contrast, rhinovirus type 2 does not induce the synthesis of LC3-positive compartments and the modification of autophagy does not result in increased viral synthesis[19]. These discrepancies led us to investigate the function of autophagy during coxsackievirus A16 infection.

In this study, we sought to clarify how the autophagy process is modulated by CA16 and to identify the molecular mechanism underlying the autophagy process. We found that CA16 infection induces the accumulation of autophagosomes. Additionally, we demonstrated that the expression of viral proteins alone could trigger incomplete autophagy. We provide the first evidence that cellular autophagy is promoted in CA16-infected cells.

## Results

### CA16 infection induces incomplete autophagy leading to autophagosome accumulation in HeLa cells

**Fig 1:**
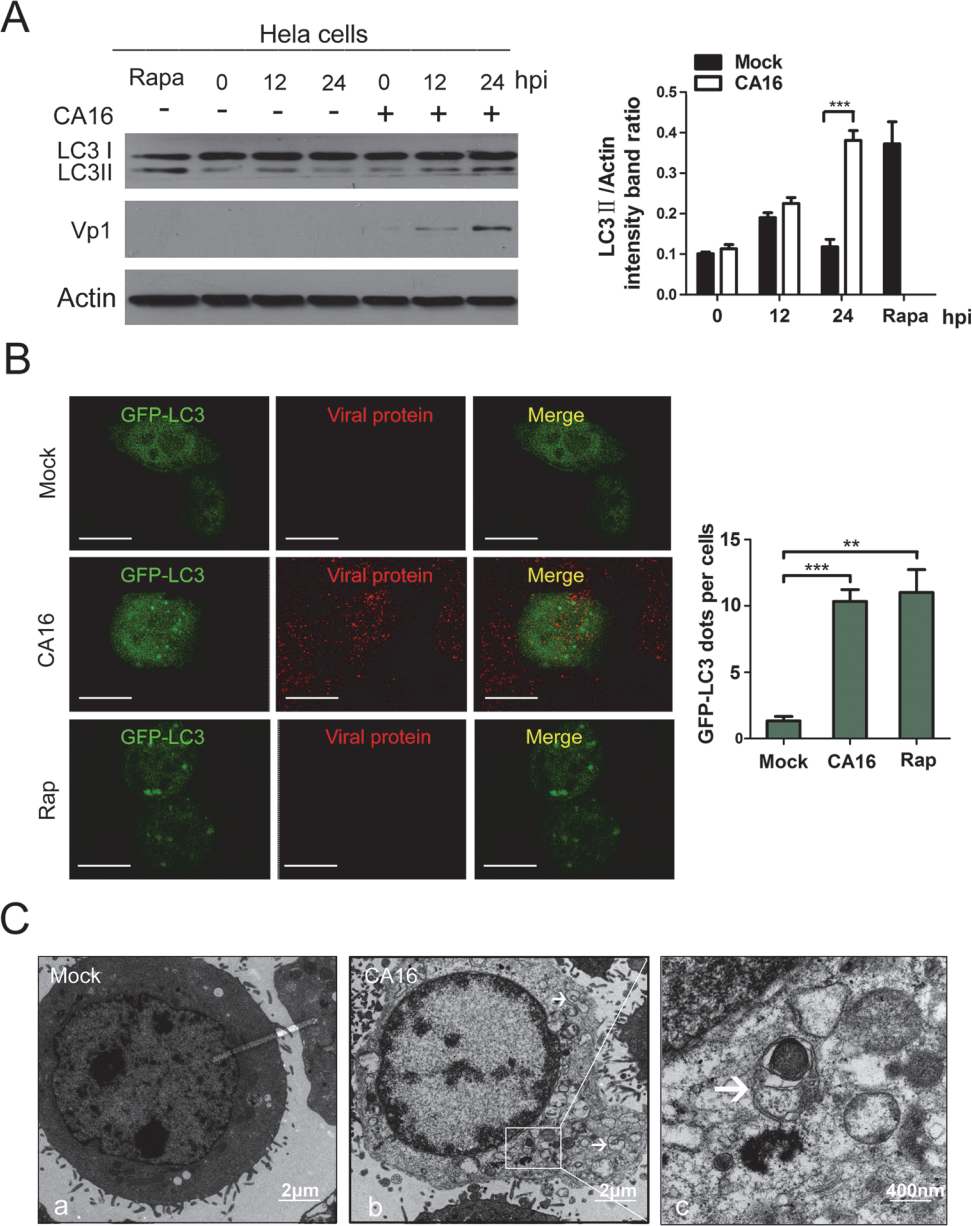
CA16 infection triggers autophagy in infected cells. (A) Western blotting (WB) analysis of LC3 protein expression in HeLa cells infected with Coxsackievirus A16 (CA16). Cells were infected with CA16 at an MOI of 1. After 1 h of virus absorption at 37°C, the cells were further cultured in maintenance medium. Cells were harvested at the indicated time points and blotted with anti-LC3B and Vp1 antibodies; the results were compared to uninfected control cells. (B) GFP-LC3 dots and viral proteins expression were visualized via confocal microscopy. HeLa cells were transfected with GFP-LC3 plasmid for 24 h, followed by CA16 infection(MOI = 1) or treated with Rapamycin and the GFP-LC3 aggregations in the cells were assessed via confocal microscopy. The localization of virus was determined by indirect immunofluorescence with a human anti-CA16 sera and TRITC-conjugated anti-human IgG. Representative images are shown. Scale bar, 8μm. (C) Autophagic vacuoles were detected via transmission electron microscopy (TEM). HeLa cells infected with CA16 (MOI = 1) were processed and analyzed at 24 hpi for the accumulation of autophagosome via electron microscopy. White arrows indicate representative autophagosomes. (c) represents the higher-magnification views of (b).

To illuminate whether CA16 could induce autophagy in infected cells, we first performed Western blotting analysis to detect the expression of LC3, which is a hallmark of autophagy. Because the LC3 precursor (LC3-I) is diffusely localized in the cytosol in resting state cells and can be quickly converted to the lipidated, autophagosome-associated form (LC3-II) after autophagy induction, the conversion of LC3-I to LC3-II is widely used to evaluate autophagic activity[9,10]. As shown in Fig 1A, the intensity of the LC3-II band was increased in CA16-infected cells relative to mock-infected cells as infection progressed. At the same time, a human polyclonal anti-CA16 serum was applied to track the progression of CA16 infection. These results showed that LC3-II levels correlated well with VP1 protein expression, indicating that the autophagy was indeed induced by CA16 infection (Fig 1A). LC3-II levels were increased in cells treated with rapamycin, a commonly used autophagy inducer. Likewise, a marked turnover of LC3-I to LC3-II was also observed in CA16-infected RD cells (S1A Fig).

Another good marker of autophagy activation is the punctate accumulation of LC3, which represents the recruitment of LC3-II to autophagic vacuoles[9,10]. To further demonstrate that CA16 infection can increase autophagosome formation, we investigated GFP-LC3 dot formation during CA16 infection. Compared with uninfected HeLa cells, large numbers of punctate GFP-LC3 proteins were observed in CA16-infected cells (P< 0.001), as evidenced by the positive red CA16 antigen staining (Fig 1B). Similar results were also observed in the rapamycin-treated group (P< 0.01) (Fig 1B). These results confirmed that CA16 infection could induce the formation of autophagosomes.

Transmission electron microscopy (TEM) is an accepted gold standard method for the identification of the morphology of autophagic compartments and the observation of the formation of double-membrane autophagic vacuoles[9,10]. Thus, we employed TEM to visually examine autophagosome formation. As demonstrated in Fig 1C, autophagic vacuoles in CA16-infected HeLa cells were significantly increased in the perinuclear region compared to uninfected cells at 24 hpi (Fig 1C–b,c). Taken together, these data strongly demonstrated that CA16 infection could induce autophagosome generation in HeLa cells.

High concentrations of drugs might influence cell viability. Because several drugs were used in this study, the CCK8 assay was performed to detect the viability of HeLa cells infected with CA16 or treated with different drugs (S2A Fig). The results indicated that the viability of HeLa cells was not obviously affected by drug treatment at the indicated doses or virus infection at the indicated time points.

**Fig 2:**
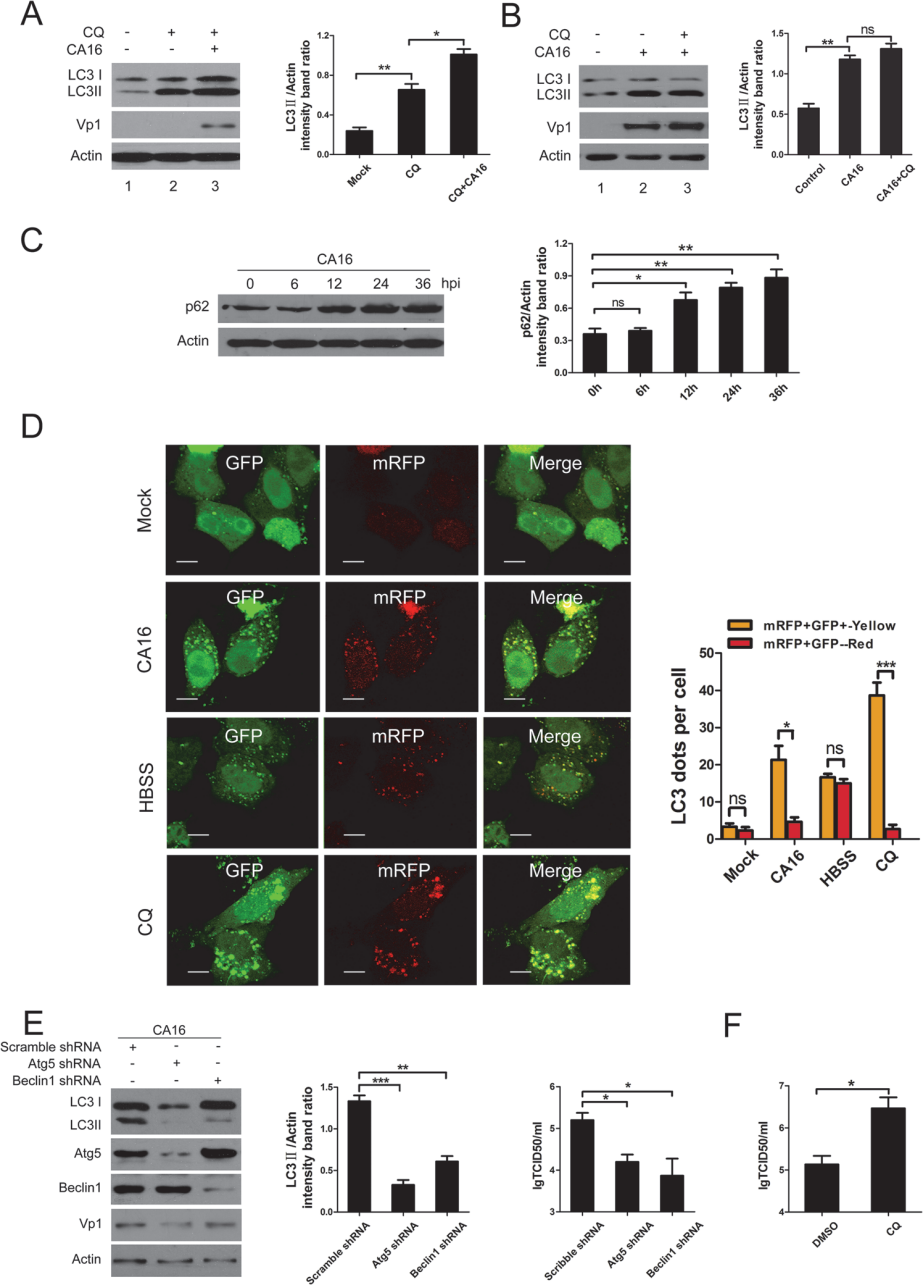
Measurement of the autophagic flux in HeLa cells infected with CA16. (A, B) Western blotting of cells with autophagy inhibited by CQ. HeLa cells were pretreated with CQ for 4 h, followed by infection with CA16 at an MOI of 2. After 1 h of virus absorption at 37°C, the cells were further cultured in maintenance medium in the absence or presence of CQ. At 24 h after infection with CA16, the cells were subjected to Western blotting using anti-LC3B and Vp1 antibodies. (C) Western blotting analysis of p62 protein expression in HeLa cells infected with CA16. Cells were infected with CA16 at an MOI of 1. After 1 h of virus absorption at 37°C, the cells were further cultured in maintenance medium. Cells were harvested at the indicated time points and detected with anti-p62 antibody compared to uninfected control cells. (D) HeLa cells transfected with ptfLC3 were infected with CA16 (MOI = 1) or treated with CQ or HBSS. The cells were collected, fixed, and visualized at 24 h postinfection. The graph shows the quantification of autophagosomes by calculating the average number of dots in 20 cells. Scale bar, 10μm. (E) Western blotting of autophagy-related proteins in cells transfected with the indicated shRNA and determination of CA16 replication in these transfected cells. HeLa cells were transfected with either specific shRNA targeting Beclin 1, Atg5 or scrambled shRNA. At 48 h after transfection, cells were infected with CA16 at an MOI of 2. Samples were collected at 12 h after infection with CA16 and detected with anti-Beclin 1,-Atg5 and-Vp1 antibodies. β-Actin was used as a protein loading control. The extracellular virus yields were determined at 48 hpi and expressed as lgTCID50/ml. Data are presented as the means from three independent experiments. Significance was analyzed with a two-tailed Student’s *t* test. *P< 0.05, **P< 0.01, ***P< 0.001. (F) Determination of CA16 replication in HeLa cells treated with CQ. HeLa cells were pretreated with CQ for 4 h followed by infection with CA16 at an MOI of 2. The extracellular virus yields were determined at 12 hpi and expressed as lgTCID50/ml. Data are presented as the means from three independent experiments. Significance was analyzed with a two-tailed Student’s *t* test. *P< 0.05.

Because autophagosomes are merely the intermediate products of the autophagy process, the accumulation of autophagosomes may be the result of either increased autophagosome biogenesis or disrupted trafficking to lysosomes for degradation. In other words, autophagosome accumulation may result from autophagy induction (completed autophagy or incomplete autophagy) or impaired autophagosome-lysosome fusion[9,10]. To clarify which of these mechanisms was responsible for the increased numbers of autophagosomes, we employed CQ (a widely used compound that prevents autophagosome-lysosome fusion by elevating the pH of the lysosomes)[10]. As shown in Fig 2A, higher levels of LC3-II accumulated in CA16-infected cells compared to mock-infected cells (Fig 2A, lanes 2 and 3) following CQ treatment, indicating that the accumulation of autophagosomes was not caused by blocking basic autophagy but by new autophagosome formation. However, no differences in LC3-II levels were observed between CA16-infected cells (moi = 2) treated with CQ compared to the untreated controls (Fig 2B, lanes 2 and 3), indicating that CA16 infection may play a similar role to CQ in blocking autophagosome maturation. Additionally, we also analyzed p62 protein levels in CA16-infected cells. P62 is another widely used autophagy flux marker because p62 links both LC3 and the ubiquitinated substrates that are degraded in the completed autophagy process after the autophagosomes fuse with lysosomes[9,10]. We failed to observe obvious degradation of p62 in CA16-infected HeLa cells even 24 hr after infection, although nearly half of the cells were severely cytopathic (S2B Fig). Inversely, higher levels of p62 were shown with the progression of CA16 infection (Fig 2C).

To further characterize whether CA16 infection impairs autophagosome maturation, we used the tandem probe ptfLC3. GFP is quenched in acidic compartments, whereas RFP retains fluorescence; thus, the fusion of autophagosomes with lysosomes will lead to the disappearance of green fluorescence, causing a fluorescence change from yellow to red and making it possible to differentiate between autophagosomes and autolysosomes[9,10]. As demonstrated in Fig 2D, large amounts of yellow autophagosomes were detected in CA16-infected cells, indicating that the autophagosomes failed to fuse with lysosomes (Fig 2D); in contrast, few yellow autophagosomes were observed in mock-infected cells. As expected, in CQ-treated cells where autophagosome and lysosome fusion was suppressed there were few red autolysosomes, but a large number of yellow autophagosomes remained detectable. Complete autophagy occurs in HBSS-treated cells [10]; these cells contained a large number of red autolysosome vacuoles (Fig 2D). Taken as a whole, our results suggest that CA16 infection induces incomplete autophagy by impairing the fusion of autophagosomes with lysosomes and blocking autophagosome maturation.

### The accumulation of autophagosomes enhances extracellular CA16 production

To analyze the effect of autophagy on CA16 replication in HeLa cells, shRNAs specifically targeting Beclin1 and Atg5 to inhibit autophagosome formation in the early stage of autophagy were applied. Beclin1, in complex with Vps34, positively regulates autophagosome formation at the nucleation step, while Atg5 conjugated to Atg12 and Atg16L1 is critical for the elongation of the phagophore membranes[9,10]. As shown in Fig 2E, both the levels of Atg5 and Beclin1 proteins were strongly reduced compared with the negative control, and the accumulation of LC3-II was suppressed by knockdown of Atg5 (P< 0.001) or Beclin1 (P< 0.01). Moreover, the suppression of Beclin1 and Atg5 expression also resulted in an evident reduction in extracellular viral production compared to the control (Fig 2E, P< 0.05). Next, we applied CQ, an acidification inhibitor of lysosome degradation that can impair autophagosome maturation in the late stage of autophagy[10]. As demonstrated in Fig 2F, viral progeny titers were significantly increased in the cells treated with CQ compared to the controls (Fig 2F, P< 0.05) at 24 hpi. Taken together, these results suggested that the accumulation of autophagosomes enhanced extracellular viral production.

### Over-expression of 2C or 3C triggers incomplete autophagy and elevates the accumulation of autophagosomes

**Fig 3:**
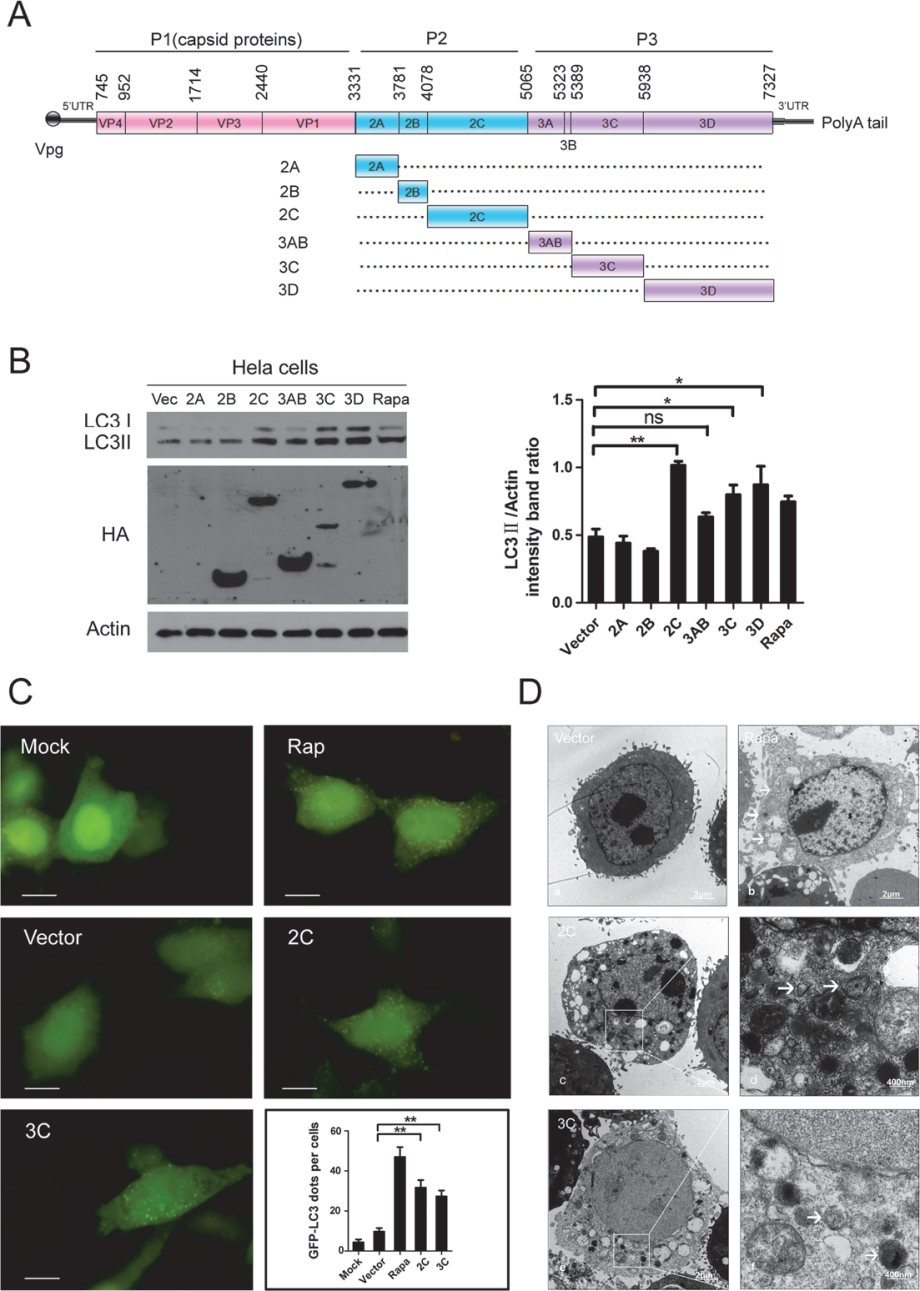
Expression of CA16 2C and 3C protein increase autophagosome accumulation. (A) Genome structure of CA16. (B) WB analysis of LC3 protein expression in HeLa cells transfected with plasmids expressing individual viral proteins. Cells transfected with the pCMV-HA empty vector or plasmids expressing CA16 non-structural proteins 2A, 2B, 2C, 3AB, 3C or 3D. Cells were harvested at 24 h after transfection, and protein expression was detected with anti-LC3B and HA antibodies. Rapamycin-treated cells were used as a positive control, and β-Actin was used as a protein loading control. Representative results are shown with graphs representing the ratio of LC3-II to β-Actin normalized to the control condition. Data are presented as the means from three independent experiments. Significance was analyzed with a two-tailed Student’s t test. *P< 0.05, **P< 0.01. (C) GFP-LC3 aggregation was visualized via fluorescence microscopy. HeLa cells were transfected with vector or HA-2C/3C plus pEGFP-LC3 for 24 h, and the GFP-LC3 aggregations in the cells were assessed via fluorescence microscopy. Representative images are shown. The number of GFP-LC3 dots in each cell was counted, and the graph shows the quantification of autophagosomes by taking the average number of dots in 20 cells. Scale bar, 10μm. (D) Autophagic vacuoles were detected via transmission electron microscopy (TEM). HeLa cells transfected with vector or viral proteins were processed and analyzed at 24 h after transfection for the accumulation of autophagosomes via electron microscopy. White arrows indicate representative autophagosomes. (d, f) represent the higher-magnification views of (c, e).

The results obtained here imply that CA16 infection resulted in autophagosome accumulation in infected HeLa cells. However, it was unclear whether viral proteins played a role in activating autophagy in CA16-infected cells. To address this question, HeLa cells were transfected with plasmids over-expressing each viral protein (2A, 2B, 2C, 3AB, 3C, and 3D, Fig 3A) of CA16. As presented in Fig 3B, scanning analysis revealed that the LC3-II/Actin ratios were higher compared to the controls in HeLa cells over-expressing the 2C (P< 0.01), 3C (P< 0.05), and 3D (P< 0.05) proteins but not the 2A, 2B, or 3AB proteins. Similar results were demonstrated in RD cells over-expressing the 2C (P< 0.001) and 3C (P< 0.05) proteins but not the 3D protein (S1B Fig). Thus, the 2C and 3C proteins with the capacity to trigger autophagosome formation in both HeLa and RD cells were chosen for further study.

To further confirm that over-expression of viral protein 2C or 3C could activate the autophagy machinery, GFP-LC3 dot formation was explored (Fig 3C). Compared with mock-infected HeLa cells, large amounts of punctate GFP-LC3 proteins were detected in cells over-expressing 2C or 3C; the quantitative assay demonstrated a higher percentage of GFP-LC3 dots in 2C or 3C over-expressing cells compared with the control cells (P< 0.01), whereas perinuclear LC3 accumulation was low in both mock and vector expressing cells (Fig 3C). Accordingly, an increase in the number of autophagosomes was also observed in HeLa cells over-expressing 2C (Fig 3D–c,d) or 3C (Fig 3D–e,f) using transmission electron microscopy. Electron microscopic examination revealed few autophagic double-layered vesicles in the vector expressing cells (Fig 3D–a) and more abundant vesicles in the cells over-expressing 2C or 3C. Taken together, our results indicated that 2C or 3C over-expression could induce the accumulation of autophagosomes. Therefore, these proteins might play an essential role in autophagy activation in CA16-infected cells.

**Fig 4:**
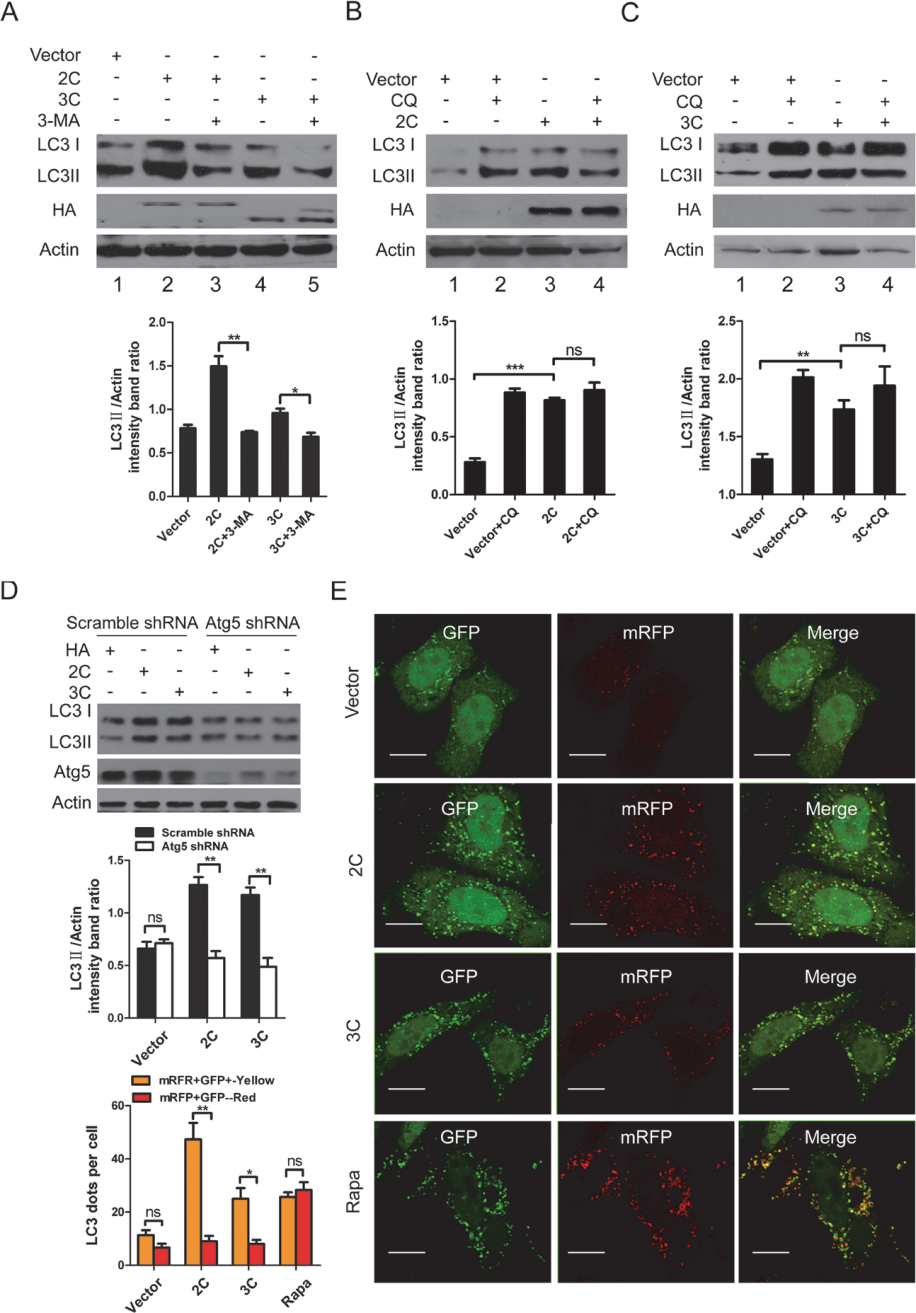
Expression of 2C and 3C failed to induce complete autophagy in HeLa Cells. (A) Western blotting of the expression levels of autophagy-related proteins in cells inhibited with 3-MA. HeLa cells were pretreated with 3-MA for 2 h, followed by transfection with viral proteins 2C and 3C. At 24 h after transfection, the cells were subjected to Western blotting using anti-LC3B and Vp1 antibodies. (B, C) Western blotting of the expression levels of autophagy-related proteins in cells inhibited with CQ. HeLa cells were pretreated with CQ for 4 h, followed by transfection with vector or viral proteins 2C and 3C in the absence or presence of CQ. At 24 h after transfection, the cells were subjected to Western blotting using anti-LC3B and HA antibodies. (D) HeLa cells were transfected with either specific shRNA targeting Atg5 or scrambled shRNA. At 12 h after transfection, cells were transfected with vector or viral proteins 2C and 3C. Samples were collected at 24 h after the second transfection and subjected to Western blotting using anti-LC3B and Atg5 antibodies. β-Actin was used as a protein loading control. Representative results are shown with graphs representing the ratio of LC3-II to β-Actin normalized to the control condition. Data are presented as the means from three independent experiments. Significance was analyzed with a two-tailed Student’s *t* test. **P< 0.01. (E) HeLa cells were cotransfected with the ptfLC3 plasmid and either an empty control vector plasmid or 2C/3C-expression plasmids and incubated for 24 h. Fluorescent signals were visualized by confocal microscopy. The graph shows the quantification of autophagosomes by calculating the average number of dots in 20 cells. Bars, 10μm.

To characterize whether viral proteins triggered autophagy via the canonical process, 3-MA (a widely used pharmacologic inhibitor of autophagy that targets Vps34 and inhibits the formation of autophagosomes at the early stage[9,10]) was applied. As demonstrated in Fig 4A, the conversion from LC3-I to LC3-II was notably suppressed in cells over-expressing 2C (P< 0.01) or 3C (P< 0.05) compared to control cells following 3-MA treatment. To rule out the nonspecific effects of the drug, we also performed a knockdown experiment to suppress the expression of Atg5, which plays a vital role in autophagosome generation[9,10]. As shown in Fig 4D, endogenous levels of Atg5 were reduced in HeLa cells transfected with shRNAs targeting Atg5 compared to control cells transfected with scrambled shRNAs. In turn, reduced Atg5 expression resulted in an approximately 2.2-fold decrease in LC3-II levels in cells over-expressing 2C or 3C compared to the control (Fig 4D, P< 0.01). These results indicated that viral proteins 2C and 3C promote autophagosome generation. Next, we treated cells with CQ to explore whether the viral proteins induced complete or incomplete autophagy. As demonstrated in Fig 4B, we found an accumulation of higher levels of LC3-II in cells over-expressing 2C compared to the control cells (Fig 4B, lanes 1 and 3, P< 0.001). However, no obvious differences in LC3-II levels were detected in cells over-expressing 2C treated with CQ compared to the untreated controls (Fig 4B, lanes 3 and 4), suggesting that 2C may function similarly to CQ and impair autophagy maturation. A similar phenomenon was also observed in CQ-treated cells over-expressing 3C (Fig 4C). To further confirm that the viral proteins may inhibit autophagosome maturation, the ptfLC3 reporter plasmid was used. As revealed in Fig 4E, HeLa cells were cotransfected with ptfLC3 and an empty vector or the 2C/3C over-expression plasmids (Fig 4E). Cells transfected with ptfLC3 and treated with rapamycin were used as a positive control. As expected, a large number of small red puncta were observed in these cells. However, in cells transfected with the ptfLC3 reporter plus HA-2C, large numbers of yellow puncta and few red autophagolysomes were detected. The number of yellow puncta in cells over-expressing 3C was also significantly increased, but red puncta were rarely seen (Fig 4E). Moreover, 2C induced the formation of more dots than 3C, consistent with the previous Western blotting results (Fig 3B and 3C). Taken as a whole, these findings suggested that either 2C or 3C could be sufficient to induce incomplete autophagy.

### CA16 infection induces autophagy potentially mediated by 2C and enhances IRGM promoter activation to increase IRGM expression

**Fig 5:**
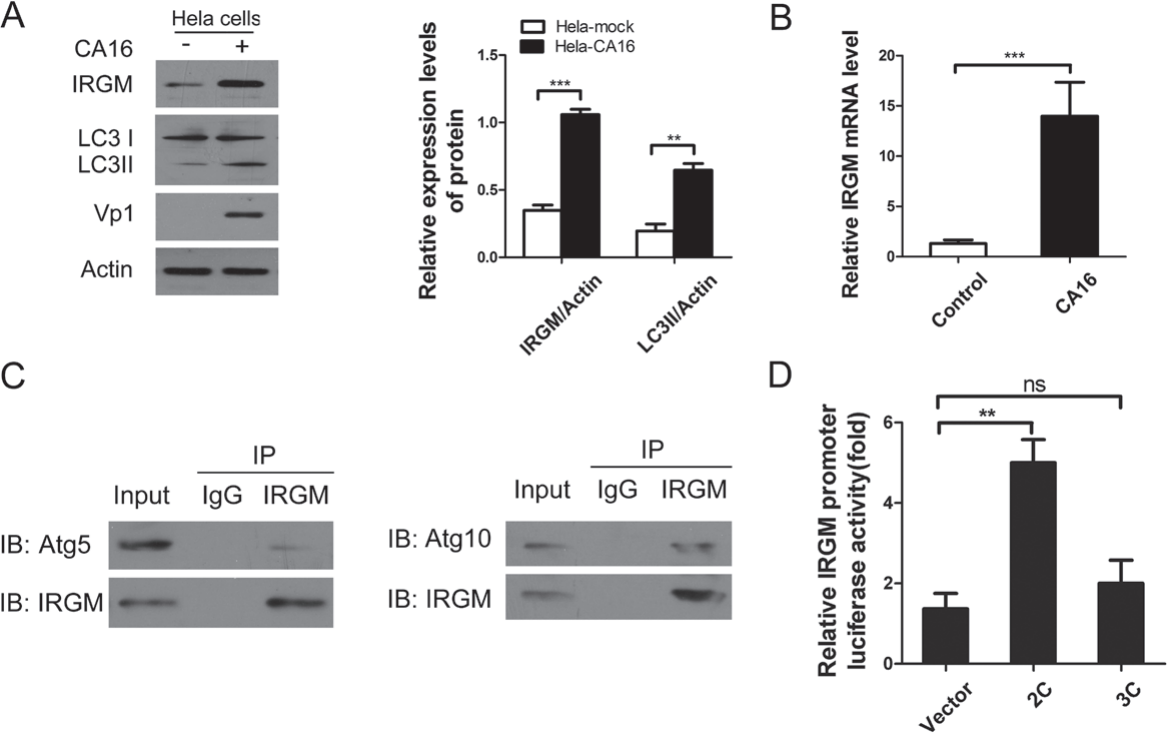
IRGM interacts with Atg5 and Atg10 and the IRGM promoter can be activated by 2C. (A) LC3-I/LC3-II and IRGM expression in HeLa cells infected with Coxsackievirus A16 (CA16). Cells were infected with CA16 at an MOI of 2, harvested at 24 hpi. Detection with anti-IRGM, LC3B and Vp1 antibodies was compared to control uninfected cells. (B) Real time-PCR analysis of IRGM mRNA levels in Hela cells that infected or not with CA16 (n = 3, ***P<0.001). (C) Endogenous IRGM interacts with endogenous Atg5 and Atg10 in CA16 infected cells. HeLa cells were infected with CA16 (MOI = 1) for 24h. Whole-cell lysates (WCL) were subjected to coimmunoprecipitation (Co-IP) with IRGM antibody or IgG, followed by SDS-PAGE/immunoblot analysis with antibodies as indicated. (D) Effects of 2C and 3C on IRGM promoter activation. HeLa cells were co-transfected with HA-2C or HA-3C and IRGM-Luc. Plasmids expressing HA and pRL-TK were used as controls. At 24 h after transfection, cells lysates were assayed for luciferase activity. Data are representative of three independent experiments with triplicate samples. **P< 0.01.

Recently, the human immunity-related GTPase family M protein (IRGM, also known as interferon-inducible protein 1 (IFI1)) was reported to be widely targeted by several RNA viruses that are capable of inducing autophagy in human cells to facilitate viral replication. However, whether and how IRGM regulates autophagy upon CA16 infection is unknown[20,21,22]. To better understand the molecular mechanism that mediates the autophagy process, we explored the relationship between IRGM expression and autophagy induction. As shown in Fig 5A and 5B, Western blotting and real-time PCR were performed and we found that CA16 infection triggered significant increases in both the mRNA (Fig 5B, P< 0.001) and protein (Fig 5A, P< 0.001) levels of IRGM. The LC3-I-to-LC3-II conversion was also notably enhanced following CA16 infection (P< 0.01). Thus, the LC3-II level positively correlated with IRGM expression (Fig 5A). Because IRGM levels increased with autophagy induction, the findings led us to question whether IRGM was able to regulate the autophagic process by interacting with certain human autophagy proteins. As confirmed by coimmunoprecipitation (Co-IP), endogenous IRGM colocalized with endogenous Atg5 (Fig 5C). Furthermore, a physical interaction between IRGM and Atg10 was also confirmed (Fig 5C). In addition, we also have assessed the exogenous Atg5-IRGM and exogenous Atg10-IRGM interaction upon CA16 infection. Similar effects were also observed (S3 Fig). Next, to evaluate whether over-expression of viral protein 2C or 3C had an effect on IRGM promoter activity, HeLa cells were cotransfected with viral protein 2C or 3C and IRGM-promoter-luc. Luciferase activity was measured 24 h after transfection. The data showed that the relative luciferase activities of the IRGM promoter were markedly promoted in cells over-expressing 2C (P< 0.01), but not in cells over-expressing 3C (Fig 5D). Additionally, we performed a Co-IP assay to detect the physical interaction between IRGM and 2C or 3C (S3C Fig). Regrettably, we failed to confirm the direct interaction between the viral proteins and IRGM. Together, our results suggested that it is possible that 2C could enhance IRGM promoter activation, resulting in increased IRGM expression and the induction of autophagy.

### IRGM is required for CA16 infection-induced autophagy

**Fig 6:**
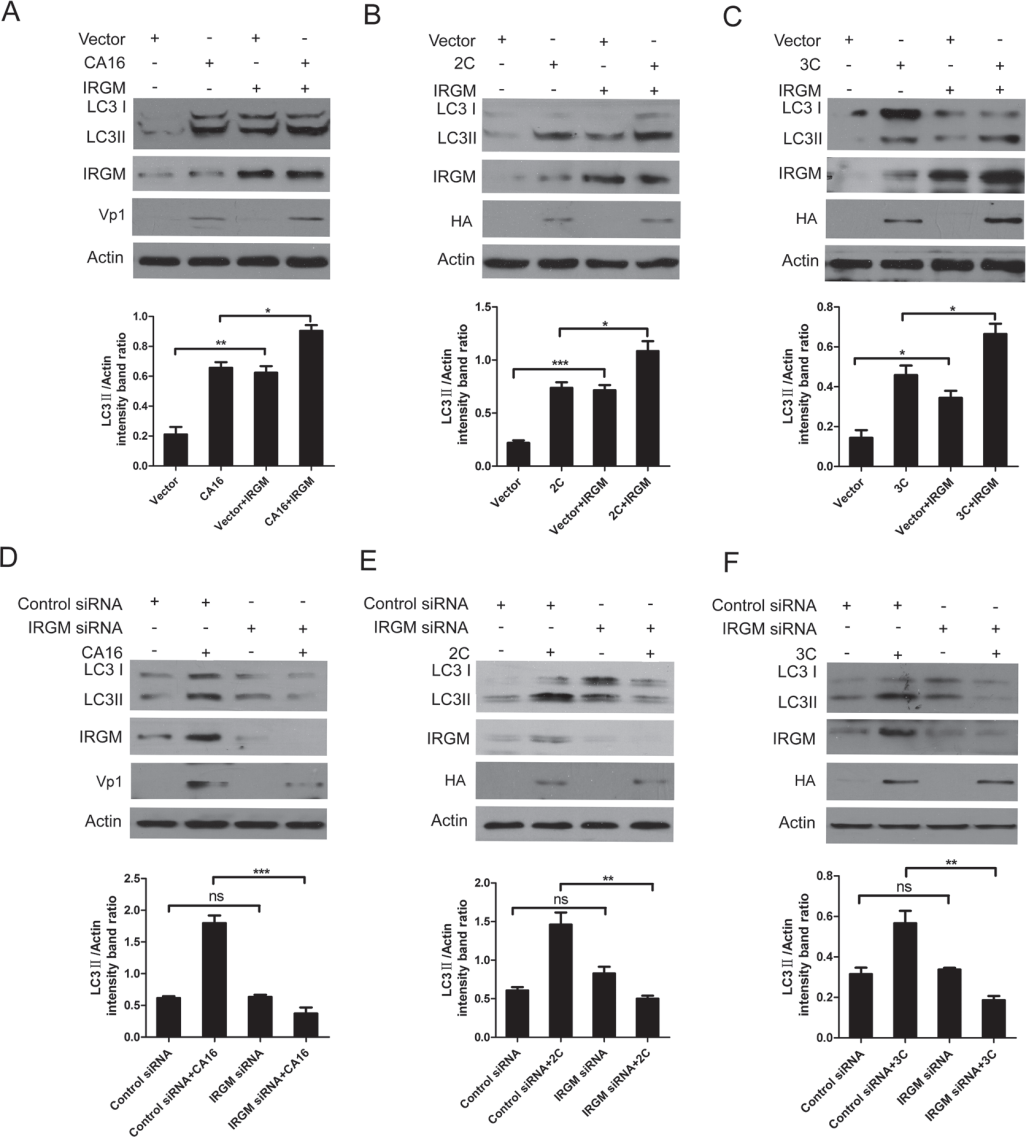
IRGM is required for both CA16 infection and 2C/3C-expression induced autophagy. (A) Overexpression of IRGM promotes CA16-mediated LC3 processing. HeLa cells were transfected with empty vector or HA-IRGM for 24 h, followed by infection with CA16 (MOI = 2). At 12 h after infection with CA16, the cells were subjected to Western blotting using anti-LC3B, IRGM and Vp1 antibodies. (B) Overexpression of IRGM promotes 2C-mediated LC3 processing. HeLa cells were cotransfected with HA-IRGM plus vector or HA-2C. At 24 h after transfection, the cells were subjected to Western blotting using anti-LC3B, IRGM and HA antibodies. (C) Overexpression of IRGM promotes 3C-mediated LC3 processing. HeLa cells were cotransfected with HA-IRGM plus vector or HA-3C. At 24 h after transfection, the cells were subjected to Western blotting using anti-LC3B, IRGM and HA antibodies. (D) Depletion of IRGM expression attenuates CA16-mediated LC3 processing. HeLa cells were transfected with siIRGM or control siRNA for 48 h, followed by infection with CA16 (MOI = 2). At 12 h after infection with CA16, the cells were subjected to Western blotting using anti-LC3B, IRGM and Vp1 antibodies. (E) Depletion of IRGM expression attenuates 2C-mediated LC3 processing. HeLa cells were transfected with siIRGM or control siRNA for 12 h, followed by transfection with vector or HA-2C. At 24 h after the second transfection, the cells were subjected to Western blotting using anti-LC3B, IRGM and HA antibodies. (F) Depletion of IRGM expression attenuates 3C-mediated LC3 processing. HeLa cells were transfected with siIRGM or control siRNA for 12 h, followed by transfection with vector or HA-3C. At 24 h after the second transfection, the cells were subjected to Western blotting using anti-LC3B, IRGM and HA antibodies. β-Actin was used as a protein loading control. Representative results are shown, with graphs representing the ratio of LC3-II to β-Actin normalized to the control condition. Data are presented as the means from three independent experiments. Significance was analyzed with a two-tailed Student’s *t* test. *P< 0.05, **P< 0.01, ***P< 0.001.

To further investigate the role of IRGM in autophagy induction, HeLa cells were transiently transfected with HA-IRGM or siIRGM to over-express or knockdown IRGM, respectively. As depicted in Fig 6A, 6B and 6C, the IRGM protein was successfully over-expressed in HeLa cells as detected by Western blotting analysis. Interestingly, there was an obvious increase in LC3-II levels in CA16-infected cells over-expressing IRGM (Fig 6A, P< 0.01). Similarly, LC3-II levels were also evidently increased in both 2C and 3C over-expressing cells (Fig 6B and 6C, *P*< 0.05) over-expressing IRGM. Next, we depleted IRGM expression using small interfering RNAs (siRNA), resulting in a marked knockdown of IRGM, as confirmed by Western blotting (Fig 6D, 6E and 6F). As shown in Fig 6D, 6E and 6F, there was a prominent decrease in LC3-II conversion in CA16-infected (P< 0.001) and 2C (P< 0.01) or 3C (P< 0.01) over-expressing cells after IRGM inhibition compared to the control. Taken together, these results indicate that IRGM is required for both CA16 infection and 2C or 3C over-expression-induced autophagy.

### CA16 modulates autophagy in part by inhibiting the Akt/mTOR/p70S6k signaling pathway and activating the MEK/ERK signaling pathway

**Fig 7:**
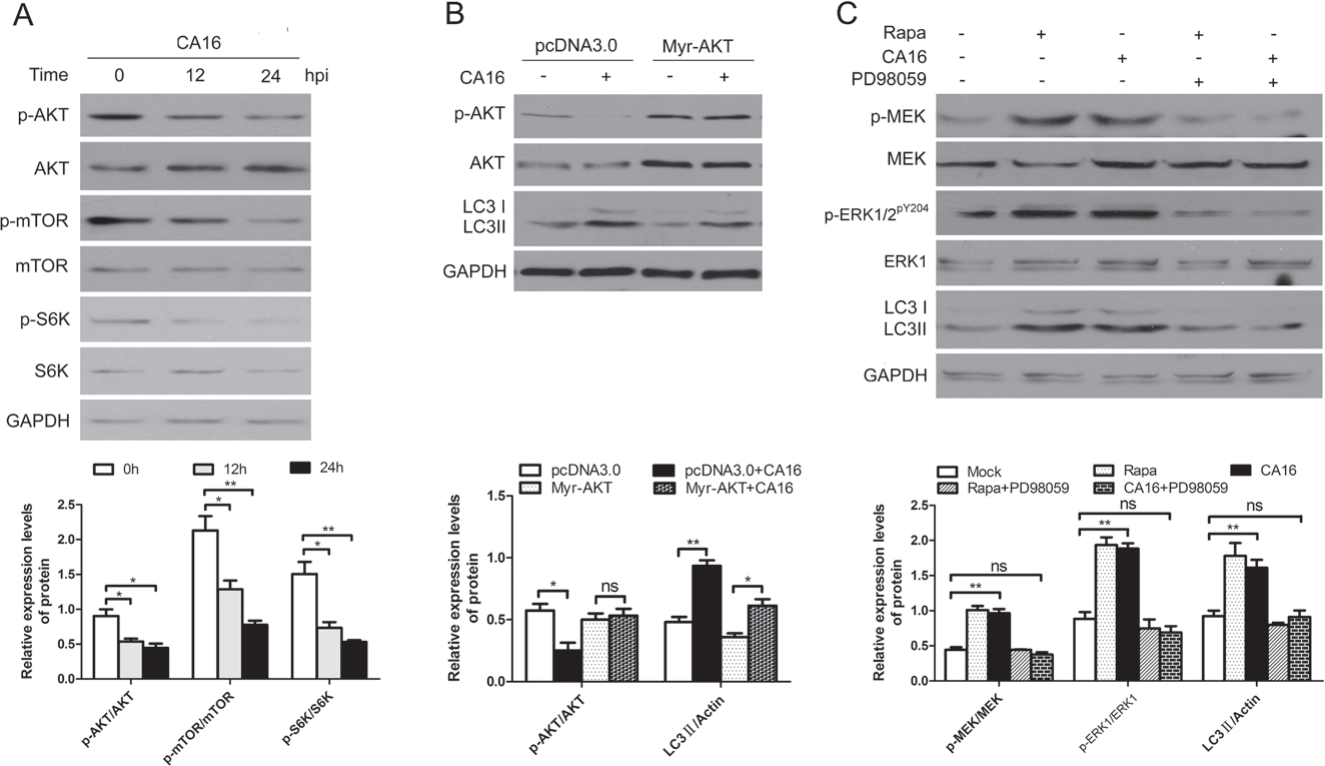
CA16 modulates autophagy in part by inhibiting the Akt/mTOR/p70S6k signaling pathway and activating the MEK/ERK signaling pathway. (A) Western blotting of Akt and p70S6k phosphorylation after CA16 infection. Cells were infected with CA16 at an MOI of 1. After 1 h of virus absorption at 37°C, the cells were further cultured in maintenance medium. Cells were harvested at the indicated time points. Protein expression was detected with the indicated antibodies and compared to uninfected controls. (B) Western blotting of CA16-induced autophagy following the overexpression of a constitutively active form of Akt (Myr-Akt). Cells were transfected with an Akt overexpression (Myr-Akt) or empty vector (pcDNA3) plasmid for 24 hours and then infected with CA16 (MOI = 1) for 12 hours. Levels of phosphorylated Akt, total Akt and LC3-II were measured by western blotting. (C) Western blotting of MEK/ERK signaling. HeLa cells were pretreated with 100 nM rapamycin (Rap) with or without 2μM PD98059 for 4 h, followed by infection with CA16 at an MOI of 2. After 1 h of virus absorption at 37°C, the cells were further cultured in maintenance medium in the absence or presence of rapamycin and PD98059. At 12 h after infection with CA16, the cells were subjected to Western blotting with the indicated antibodies. GAPDH was used as the loading control. Data are presented as the means from three independent experiments. Significance was analyzed with a two-tailed Student’s *t* test. *P< 0.05, **P< 0.01, ***P< 0.001.

Both the Akt/mTOR and MEK/ERK signaling pathways have been reported to regulate various aspects of cell physiology, including autophagy[11,12,23,24]. To explore whether Akt/mTOR signaling is involved in CA16 infection-induced autophagy, we evaluated the phosphorylation of these kinases. As shown in Fig 7A, CA16 infection inhibited the phosphorylation of Akt at 12 and 24 hpi but had no obvious effect on total Akt levels in HeLa cells. Additionally, the levels of ribosomal protein p70S6 kinase (p70S6k) phosphorylation (a well-known mTOR substrate that is phosphorylated by mTOR) were detected to evaluate mTOR kinase activity. As depicted in Fig 7A, p70S6 kinase phosphorylation was notably suppressed at 12 and 24 hpi. To explore whether Akt/mTOR signaling was involved in autophagy induction, a plasmid expressing the myristoylated and constitutively active form of the Akt1 protein (Myr-Akt) was applied. As expected, over-expression of activated Akt prevented the decrease of phosphorylated Akt levels and disrupted CA16-induced autophagy (Fig 7B). Next, we examined MEK/ERK levels in response to autophagy stimuli after CA16 infection to clarify the role of MEK/ERK in the induction of autophagy. As demonstrated in Fig 7C, both rapamycin and CA16 infection caused autophagic responses, as confirmed by higher levels of LC3-II expression. Moreover, the levels of MEK/ERK phosphorylation were also increased. Finally, the MEK inhibitor PD98059 was used to test whether the autophagy induction was dependent on MEK/ERK activation. As expected, disrupting MEK/ERK with PD98059 blocked the autophagic response. Taken together, our results indicated that CA16 triggered autophagy in part by modulating the Akt/mTOR/p70S6k and MEK/ERK signaling pathways.

## Discussion

In this paper, we report for the first time that CA16 infection increases autophagosome accumulation in HeLa cells in a manner that facilitates virus production. We also show that the expression of the viral 2C protein enhances autophagosome accumulation by blocking autophagosome-lysosome fusion in an IRGM-dependent manner. Moreover, we demonstrate that CA16 modulates autophagy, in part, by inhibiting the Akt/mTOR/p70S6k signaling pathway and activating the MEK/ERK signaling pathway.

Because autophagy is part of the cellular intrinsic immune system, a growing number of studies have reported different strategies that have been evolved by viruses to impair or trigger autophagy for their own benefit[25,26]. Some viruses have been shown to inhibit autophagic processes to avoid immune defense or degradation. For example, human papillomavirus type 16[11], human cytomegalovirus (HCMV)[27], and Kaposi’s sarcoma-associated herpesvirus (KSHV)[28] have been demonstrated to inhibit cellular autophagy through different pathways in infected cells. In addition to autophagy inhibition, a large numbers of viruses have been reported to promote autophagy. For example, hepatitis C virus (HCV)[29]and human T-cell leukemia virus type 1 (HTLV-1)[30] infection can trigger autophagy. Dengue virus (DENV)[17], hepatitis B virus (HBV) [31] and influenza A virus (IAV)[32] not only have been shown to induce autophagy but also upregulate autophagy to promote virus replication. Interestingly, human parainfluenza virus type 3 (HPIV3)[33], rotavirus and human immunodeficiency virus type 1(HIV-1)[34] have been reported to induce autophagy but to block the fusion between autophagosomes and lysosomes, leading to autophagosome accumulation to facilitate viral production. In this study, we found that CA16 infection could induce autophagy. However, the autophagosomes failed to fuse with the lysosomes, leading to significant autophagosome accumulation.

The available data indicate that the accumulated autophagosomes may in turn favor the reproduction of RNA viruses by several mechanisms. First, autophagosomes that fail to fuse with lysosomes may prevent the newly formed virions or viral RNA from degrading or being processed by autolysosomes or Toll-like receptor 7 (TLR-7), which is present in late endosomes and lysosomes[13,14,15]. Moreover, autophagy related proteins may also be involved in the innate immune response to virus replication. For example, Atg5-deficient mouse embryonic fibroblasts (MEFs) were resistant to vesicular stomatitis virus replication, largely resulting from an Atg12-Atg5 conjugate that negatively regulated type I IFN production[35]. Moreover, knockdown of beclin1 has been reported to suppress hepatocytes[36]. In this study, we found that deletion of Agt5 and Beclin1 downregulated extracellular virus titers. Therefore, it is tempting to speculate that in addition to influencing autophagy, Agt5 and Beclin1 may also benefit viruses by regulating cellular immune processes. The autophagosome provides a physical scaffold where the viral replication machinery can be assembled. Decades ago, Schlegel S. et al. reported that poliovirus infection caused a massive rearrangement of intracellular membranes, with a large number of double-membraned vesicles accumulating in the cytoplasm[37]. Subsequent research further suggested that the cytoplasmic surfaces of these membranous vesicles were the sites of viral RNA replication[38]. In recent years, dengue virus has been reported to co-localize with autophagy markers, suggesting that these viruses might assemble on autophagic vesicles, thereby subverting the autophagy machinery for their benefit[17,38]. Similarly, we found that CA16 infection triggered autophagosome accumulation that facilitated extracellular virus production. When autophagosome generation was suppressed or promoted using small interfering RNA targeting Atg5 or employing CQ, respectively, the extracellular virus levels were suppressed or promoted correspondingly (Fig 2E and 2F). The positive correlations between the decreased accumulation of autophagosomes and decreased extracellular viral yields led us to speculate that the vast cytoplasmic membrane provides sites for viruses to adhere and replicate in a manner similar to poliovirus and dengue virus.

Many viral proteins have been demonstrated to play important roles in the regulation of autophagy. For example, nonstructural proteins 2C and 3D of encephalomyocarditis virus can trigger the formation of autophagosomes by activating the ER stress pathway in BHK-21 cells[39]. A previous study showed that nonstructural proteins 2BC and 3A of poliovirus were sufficient to induce double-membraned vesicles, which in turn provided membranous supports for viral RNA replication and enabled the nonlytic release of progeny virions from infected cells[40]. Interestingly, the expression of 2C or 2BC of poliovirus was revealed to be capable of inducing the proliferation of vacuoles that were morphologically similar to those found during poliovirus infection[41]. Although the relationship between a few viral proteins and the occurrence of autophagy have been demonstrated, no viral protein from coxsackievirus has been reported to induce autophagy. In this study, we expressed all of the CA16 nonstructural proteins and found that the independent expression of the 2C and 3C proteins was sufficient to induce autophagy; in turn, this process may also be synergistically influenced by other viral proteins.

Previous studies demonstrated that viral proteins may modulate autophagy by targeting certain autophagy-related proteins, such as immunity-related GTPases (IRG proteins) and especially family M proteins (IRGM)[22]. IRG is an early resistance system targeting intracellular pathogens and can be found as multiple tandem copies in the genomes of most mammalian species[20,21,22]. There are 23 complete Irg genes in the mouse genome, whereas only 2 identifiable IRG genes are found in the human genome (IRGC and IRGM)[20,21,22]. Human IRGC is very similar to mouse IRGC and is expressed in the testis; the human IRGM protein is also expressed in some cultured cells including macrophage and HeLa cells[20,21,22]. IRGM is known to play a protective role against bacterial infections, such as favoring the elimination of *Mycobacterium bovis* in macrophages and adherent-invasive *Escherichia coli*[13,14]. Another study significantly enriched the functions of IRGM and suggested that IRGM is a common target of RNA viruses that subvert the autophagy network. This targeting is accomplished by directly interacting with autophagy-associated proteins that are necessary for autophagosome formation during several virus infections, including measles virus (MeV), hepatitis C virus (HCV) and human immunodeficiency virus-1 (HIV-1)[22]. However, whether IRGM also played a key role in picornaviridae-induced autophagy remained unknown. In this study, we found that IRGM was upregulated in CA16-infected cells and that this upregulation was accompanied by a significant autophagic response. In contrast, autophagy was suppressed in IRGM knockdown (siIRGM) cells. Thus, IRGM is crucial for the activation of CA16 infection-induced autophagy, which is in line with previous reports. Strikingly, the promoter of IRGM was targeted by 2C to enhance IRGM expression but was not by 3C. Although both 2C and 3C are non-structural proteins which play key roles in the replication of picornaviruses, they function differently. 2C is an ATPase that plays a key role in host cell membrane rearrangement, is a putative helicase, and is implicated in virion assembly and packaging[42,43]. In addition to its NTPase and RNA binding activities, 2C is also endowed with the capacity to induce the formation of cytoplasmic vacuoles morphologically similar to those found during virus infection[44]. However, the picornavirus 3C are substrate-specific proteases which perform multiple tasks in viral precursor cleavage and pathogen–host interactions and previous results have suggested that both the basal transcription factor TBP (TATA-binding protein) and transcription activator proteins such as CREB (cyclic AMP-responsive element-binding protein) and Oct-1 (the octamer-binding factor) are cleaved by the viral-encoded protease, 3C(Pro)[45]. Previous findings also show that 3C(pro) plays a major role in processing poly(A)-binding protein (PABP) during virus infection which has been proposed to contribute to host translation shutoff[46]. Therefore, it is tempting to speculate that 2C and 3C may regulate IRGM in different ways leading to 2C activated IRGM luc activity directly and 3C regulate the expression of IRGM indirectly.

Recently, a growing number of studies have shown that viruses can regulate autophagy via different signaling pathways. For example, hepatitis C virus (HCV) can induce autophagosome formation by inducing endoplasmic reticulum stress[47]. Herpes simplex virus type 1 (HSV-1) triggers autophagy through the protein kinase R (PKR)/eIF2α signaling pathway[48]. Epstein-Barr virus modulates autophagic activation via the extracellular signal-regulated kinase (ERK) pathway[49]. The mammalian target of rapamycin (mTOR) is one of most characterized mediators that negatively modulates autophagosome formation. Some viruses have been reported to regulate mTOR signaling to control autophagy, such as hepatitis E virus, influenza A virus and West Nile virus[23,50,51]. Although some picornaviruses have been reported to regulate autophagy, the detailed molecular mechanism of autophagy induction and the role of the related signaling pathway remains largely unknown. Here, we found that AKT was inhibited by CA16 infection, contributing to the upregulation of autophagy. Furthermore, expression of constitutively active myr-Akt suppressed autophagy, demonstrating that CA16 infection triggers autophagy via a mechanism that involves, in part, the regulation of Akt function. The ERK signaling cascade is another vital pathway that plays a significant role in the regulation of various cellular processes, such as proliferation, differentiation and apoptosis; its inappropriate activation is a common occurrence in human cancers[12]. Thus, various steps in this signaling pathway have been targeted for therapeutic intervention. However, studies addressing whether and how the ERK cascade regulates autophagy (especially virus infection-induced autophagy) remain very limited. In this study, we verified that ERK activation is necessary for CA16 infection-induced autophagy, but the relationship between this pathway and the autophagy machinery needs to be further validated.

It is interesting to understand the relationship among IRGM, mTOR, and MAPK activities which participate in the molecular regulation of autophagy. Akt signaling is known to negatively regulate autophagy via activation of target of rapamycin (mTOR), which inhibits autophagy initiating kinases via phosphorylation[9,10,11]. ERK also has been reported to be implicated in autophagy regulation[12]. Furthermore, previous findings show that growth factor stimulation may result in the p-ERK colocalized with autophagosomal (LC3-II) and preautophagosomal (ATG5-ATG12 and ATG16) structure[52]. In the meantime, ERK and its upstream kinase MEK localize to the extraluminal face of autophagosomes[52]. IRGM also has been confirmed to be associated to mitochondria and interact with autophagy-associated proteins (ATG5) directly upon virus infection[22]. Based on previous findings and our results, it is attractive to speculate that upon CA16 infections Akt signaling may be inhibited, which play vital roles in autophagy initiation. Then IRGM interacts/recruits its protein partners at the mitochondria to induce autophagosomes formation especially as mitochondria is one possible source of membrane for autophagosome biogenesis[21]. Then the ATG5-ATG12-positive preautophagosomes and LC3-II positive membranes serve as scaffolds or cellular signaling platforms that facilitate efficient spatial coordination of the Raf-MEK-ERK cascade and thus facilitate growth factor-induced ERK phosphorylation[52]. Of course all these hypotheses need to get the experiment certification further.

Taken together, the present study confirms that CA16 triggers incomplete autophagy to enhance virus replication. Importantly, for the first time, we identified viral proteins 2C and 3C of CA16 as being sufficient for the induction of incomplete autophagy. We demonstrated that inhibition of the Akt/mTOR/p70S6k signaling pathway and activation of the MEK/ERK signaling pathway are critical for autophagy induction. Our study provides insights into CA16-host interactions and increases our understanding of the roles of autophagy and autophagy-related regulation in the virus life cycle, thereby raising the possibility of developing more specific antiviral treatments.

## Materials and Methods

### Ethics statement

This study has been approved by the ethics committee at the School of Medicine, Wuhan University, in accordance with the guidelines for the protection of human subjects. Written informed consent was obtained from the parents of all the patients involved in our study.

### Cells, viruses and plasmids

RD (rhabdomyosarcoma) and HeLa cells were purchased from the American Type Culture Collection (ATCC). RD cells were maintained in minimum essential medium (MEM) supplemented with 10% fetal bovine serum (FBS) (SV30087; HyClone) or 2% FBS (maintenance medium). HeLa cells were cultured in Dulbecco’s modified Eagle’s medium (DMEM) with 10% FBS or 2% FBS. The cells were cultured at 37°C in a humidified incubator with 5% CO2. CA16 is a laboratory strain that has been completely sequenced and belongs to the B1 genotype. The following plasmids were constructed by our laboratory: cDNA encoding LC3 was cloned into pEGFP-N1 (Clontech); cDNAs encoding CA16-2A,2B,2C,3AB,3C,3D, Atg5 and Atg10 were cloned into pCMV-HA (Clontech); IRGM were cloned into both pCMV-HA (Clontech) and pCMV-Myc (Clontech); double-stranded oligonucleotides corresponding to the Atg5 and Beclin1 target sequences were cloned into pLKO.1-TRC (Addgene); and DNA encoding the IRGM promoter region was cloned into the pGL3-basic vector (Promega). The primers and sequences are shown in S1 Table. The tandem fluorescent monomeric red fluorescent protein mRFP-GFP-LC3 (ptfLC3), constitutively active Akt (Myr-Akt1) and empty vector (pcDNA3) plasmids were purchased from Addgene.

### Reagents and antibodies

Rapamycin (Rapa), chloroquine (CQ) and 3-methyladenine (3-MA) were purchased from Sigma-Aldrich. PD98059 was purchased from Selleck. Hank's Balanced Salt Solution (HBSS) was purchased from HyClone. Rabbit anti-LC3B, anti-Atg5, anti-Atg10, anti-Beclin1, anti-p62/SQSTM1, non-phospho- or phospho- mTOR, S6K and AKT polyclonal antibodies (Abs) were purchased from Cell Signaling Technology. Non-phospho- or phospho-MEK1/2, non-phospho-ERK1, phospho-ERK1/2^pY204^ polyclonal Abs, anti-Actin and anti-GAPDH Abs were purchased from Abcam. Rabbit anti-IRGM was obtained from Abgent. Anti-HA mAb was purchased from Abmart. TRITC-conjugated goat anti-human antibody was obtained from Proteintech Group. One human polyclonal anti-CA16 serum was applied. IRGM small interfering RNAs (siRNAs) and the control scrambled siRNA were designed and synthesized by GenePharma.

### Viral infection, drug treatment and cell viability assay

HeLa and RD cells were seeded into 6-well or 12-well plates and cultured until 80% confluency was reached. Then, the cells were infected with CA16 at a multiplicity of infection (MOI) of 1 or 2. After 1 hour, the cells were washed three times with phosphate-buffered saline (PBS) to remove unattached viruses and were incubated in maintenance medium (2% FBS) at 37°C for the indicated times. HeLa cells were pretreated with optimal concentrations of rapamycin (Rapa, 100 nM) or chloroquine (CQ, 100μM) for 4 h and then infected with CA16 as described above. For 3-MA (10 mM) treatment, cells were pretreated for 2 hr and then treated again following CA16 infection until the samples were harvested. Virus titers were calculated as 50% tissue culture infectious doses (TCID50) using the Reed-Münch method[53]. The CCK8 assay kit (Beyotime) was used to evaluate cell viability as described previously[6].

### Transmission electron microscopy (TEM)

HeLa cells infected with CA16 (MOI = 1) or transfected with 2C or 3C were collected at 24 h postinfection or transfection, respectively. Cells were fixed with 2.5% glutaraldehyde overnight and subjected to preparation for TEM observation[54]. Autophagosomes were defined as double- or single-membrane vesicles measuring 0.3 to 2.0μm in diameter.

### Confocal fluorescence microscopy

HeLa cells were seeded in 12- or 24-well plates that contained coverslips and were grown to 70% confluence. Then, HeLa cells were transfected with GFP-LC3 or ptfLC3 using Lipofectamine 2000 (Invitrogen) according to the manufacturer’s guidelines. The cells were treated with virus infection or drugs as described above at 24 h post-transfection. Treated cells were washed twice with PBS and fixed in 4% paraformaldehyde in PBS for 15 min. Coverslips were inverted onto slides containing 50% glycerol, and fluorescence signals were visualized with a confocal fluorescence microscope or fluorescence microscope.

### Western blotting

Cells infected with CA16 or treated with drugs were washed twice with PBS and lysed on ice with RIPA buffer (Beyotime technology) containing a 1× protease inhibitor cocktail. Then, the lysates were centrifuged for 10 min at 13,000 rpm at 4°C to clear cellular debris deposits. The samples were boiled at 100°C for 5 min in sample buffer (2% SDS, 10% glycerol, 60 mM Tris [pH 6.8], 5% β-mercaptoethanol, and 0.01% bromophenol blue) before electrophoretic separation. The lysates were resolved by sodium dodecyl sulfate-polyacrylamide gel electrophoresis (SDS-PAGE) and transferred to polyvinylidene fluoride (PVDF) membranes (Millipore, Bedford, MA, USA). The membranes were incubated in blocking buffer (5% nonfat milk powder in Tris-buffered saline containing 0.1% Tween 20 [TBST]) for 2 h at room temperature. Next, the membranes were reacted with primary antibodies overnight at 4°C followed by horseradish peroxidase (HRP)-conjugated secondary antibody (Tianjin Sungene Biotech Co, Ltd) for 1.5 h at room temperature. Antibody-antigen complexes were observed using enhanced chemiluminescence (Pierce, Rockford, IL, USA) with a Kodak imager (Carestream Health). Band intensities were quantified using ImageJ software (NIH, Bethesda, MD).

### Coimmunoprecipitation (co-IP)

HeLa cells were infected with CA16 or transfected with the indicated plasmids using Lipofectamine 2000 (Invitrogen) according to the manufacturer’s instructions. Cells were washed twice with PBS and lysed on ice for Western blotting and IP with cell lysis buffer (Beyotime technology) containing a 1× protease inhibitor cocktail. The samples were collected by centrifugation at 13,000 rpm for 10 min at 4°C and precleared by incubation with protein G Sepharose beads (Abmart) for 3 hr at 4°C with rotation. Following centrifugation, primary antibodies were added to the supernatants and incubated for 4 hr at 4°C with rotation, and then, protein G Sepharose beads were added and incubated overnight at 4°C with rotation. Finally, the beads were collected and washed three times with washing buffer (5% sucrose, 5 mM Tris-HCl [pH 7.5], 5 mM EDTA, 500 mM NaCl, and 1% Triton X-100). The beads were denatured at 100°C for 5 min in 2×SDS protein loading buffer and analyzed by WB.

### Immunofluorescence analysis

Cell monolayers prepared on glass coverslips were fixed for 10min at room temperature in 4% paraformaldehyde (PFA) in PBS and then washed three times with PBS. Next cell permeabilization was performed by incubating the coverslips for 4 min with 0.5% Triton X-100. The cells were stained with polyclonal anti-CA16 serum overnight at 4°C, followed by a TRITC-conjugated goat anti-human antibody (1:100). Coverslips were mounted with 50% Glycerol before being analyzed on a confocal fluorescence microscope.

### RNA Extraction and Real-time PCR

According to the manufacturer’s guidelines, total RNA was extracted using Trizol reagent (Invitrogen). Then, 2 μg total RNA was reverse transcribed into 20ul cDNA using Reverse Transcription kit (Thermo) according to the manufacturer’s protocol. cDNA samples were subjected to real-time PCR(Bio-Rad iQ5) by using SYBR green (Gene Copoeia Inc.). GAPDH was used as an internal control for the expression of IRGM mRNA. The primers used are shown in S1 Table. To determine the IRGM mRNA levels, the following conditions were used: 94°C for 5 min, followed by 40 cycles of 94°C for 10 s, 55°C for 20 s and 72°C for 30 s. All samples were run in triplicate and data analysis was performed using the 2^−ΔΔCt^ method.

### RNA interference

HeLa cells were plated in 6-well or 12-well plates 24 hrs prior to transfection. When the cells reached 50% confluency they were transiently transfected with various siRNAs at a concentration of 50 to 80 nm using Lipofectamine 2000 (Invitrogen) according to the manufacturer’s instructions. Scrambled siRNA was used as the negative control. The silencing efficiency was determined by Western blotting using the respective antibodies.

### Luciferase reporter assay

Luciferase assays were conducted with a dual-luciferase reporter assay system kit (Promega, Madison, WI, USA) according to the manufacturer’s protocol. Briefly, HeLa cells were seeded into 24-well plates, and the luciferase reporter plasmid containing the IRGM promoter region was cotransfected with pLR-TK (Promega) and 2C or 3C. Twenty-four hours later, the cells were collected and washed once with PBS. Next, passive lysis buffer (Promega) was added to the cells. After 15 min, supernatants were collected following centrifugation at 12,000xg for 30 s, and the relative luciferase expression levels were analyzed using the Modulus single-tube multimode reader (Promega).

### Statistical analysis

Data were expressed as the means±standard deviations. Statistical analyses were performed using GraphPad Prism (GraphPad Software, La Jolla, CA, USA) to evaluate the differences between experimental groups. Statistical significance was determined using Student’s *t*-test and expressed as *p*-values. *p*<0.05 was considered to be statistically significant.

## Supporting Information

S1 Fig CA16 infection and viral proteins 2C, 3AB and 3C triggers autophagy in RD cells. (A) Western blotting(WB) analysis of LC3 protein in RD cells infected with CA16. Cells were infected with CA16 or not at an MOI of 0.1 and after 1 h of virus absorption at 37°C, the cells were further cultured in maintain medium. Cells were harvested at 12h after infection and detected with anti-LC3B and Vp1 antibodies. (B) Western blotting(WB) analysis of LC3 protein in RD cells transfected with plasmids expressing individual virus proteins. Cells transfected with pCMV-HA empty vector or plasmids expressing non-structure proteins 2A, 2B, 2C, 3AB, 3C or 3D of CA16. Cells were harvested at 24 h after transfection and detected with anti-LC3B and HA antibodies. Rapamycin-treated cells were used as positive control and β-actin was used as a protein loading control. Equal amounts of each cellular samples were loaded in each well of the gels. Representative results are shown with graphs representing the ratio of LC3-II to β-actin normalized to the control condition. Data are presented as means from three independent experiments. Significance was analyzed with two-tailed Student’s t test. *P< 0.05, **P< 0.01, ***P< 0.001.
(TIF)

S2 Fig CA16 infection or drugs treatment has no effect on viability of infected cells and the morphology of the infected Hela cells at different time. (A) Hela cells were infected with CA16 (MOI = 2) for 12h or were treated with optimal concentrations of rapamycin (Rapa, 100 nM), 3-methyladenine (3-MA, 5 mM) or chloroquine (CQ, 50 uM) for 24h and then cells viability was assessed by CCK8 analysis. All data are representative of at least three independent experiments, with each measurement performed in triplicate (mean ± SD of fold-change). *P< 0.05, **P< 0.01, ***P< 0.001. (B) The morphology of the infected Hela cells was investigated by microscopy at the indicated time points. Cells were infected with CA16 or not at an MOI of 0.1 and after 1 h of virus absorption at 37°C, the cells were further cultured in maintain medium. Cytopathic effect were observed at the indicated time points.
(TIF)

S3 Fig Exogenous IRGM interacts with exogenous Atg5 (A) and Atg10 (B), but IRGM failed to interact with 2C and 3C directly. HeLa cells were co-transfected with HA-Atg5 (A) or HA-Atg10 (B) and Myc-IRGM for 24 h, followed by CA16 infection (MOI = 2) for 12h. Cell lysates were subjected to immunoprecipitation using anti-HA antibody followed by WB analysis with anti-HA and anti-Myc antibodies. (C) Hela cells were transfected with a vector, HA-2C or HA-3C constructs. Whole-cell lysates (WCL) were subjected to IP with anti-HA antibody, followed by WB with IRGM and HA antibodies.
(TIF)

S1 Table Primers used for the construction of various plasmids and qRT-PCR. Bold And Italics, restriction endonuclease cutting sites.
(DOC)
