# Supplementary figures and images for: Coxsackievirus A16 Elicits Incomplete Autophagy Involving the mTOR and ERK Pathways
Source: PLoS One. 2015 Apr 8;10(4):e0122109. doi: 10.1371/journal.pone.0122109 (PMC4390341; doi:10.1371/journal.pone.0122109)

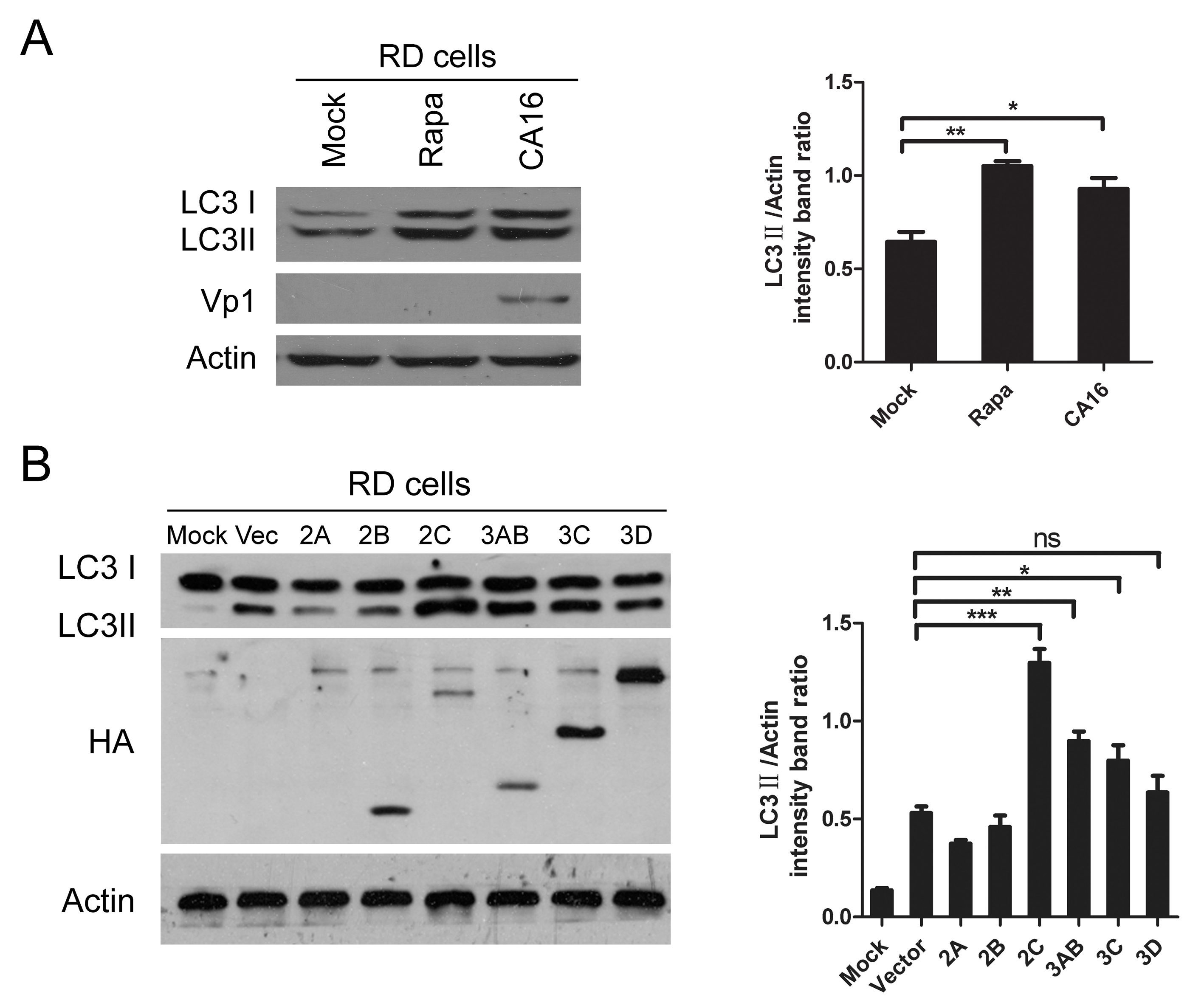

Supplement: S1 Fig — (A) Western blotting(WB) analysis of LC3 protein in RD cells infected with CA16. Cells were infected with CA16 or not at an MOI of 0.1 and after 1 h of virus absorption at 37°C, the cells were further cultured in maintain medium. Cells were harvested at 12h after infection and detected with anti-LC3B and Vp1 antibodies. (B) Western blotting(WB) analysis of LC3 protein in RD cells transfected with plasmids expressing individual virus proteins. Cells transfected with pCMV-HA empty vector or plasmids expressing non-structure proteins 2A, 2B, 2C, 3AB, 3C or 3D of CA16. Cells were harvested at 24 h after transfection and detected with anti-LC3B and HA antibodies. Rapamycin-treated cells were used as positive control and β-actin was used as a protein loading control. Equal amounts of each cellular samples were loaded in each well of the gels. Representative results are shown with graphs representing the ratio of LC3-II to β-actin normalized to the control condition. Data are presented as means from three independent experiments. Significance was analyzed with two-tailed Student’s t test. *P< 0.05, **P< 0.01, ***P< 0.001. (TIF) [file pone.0122109.s001.tif]

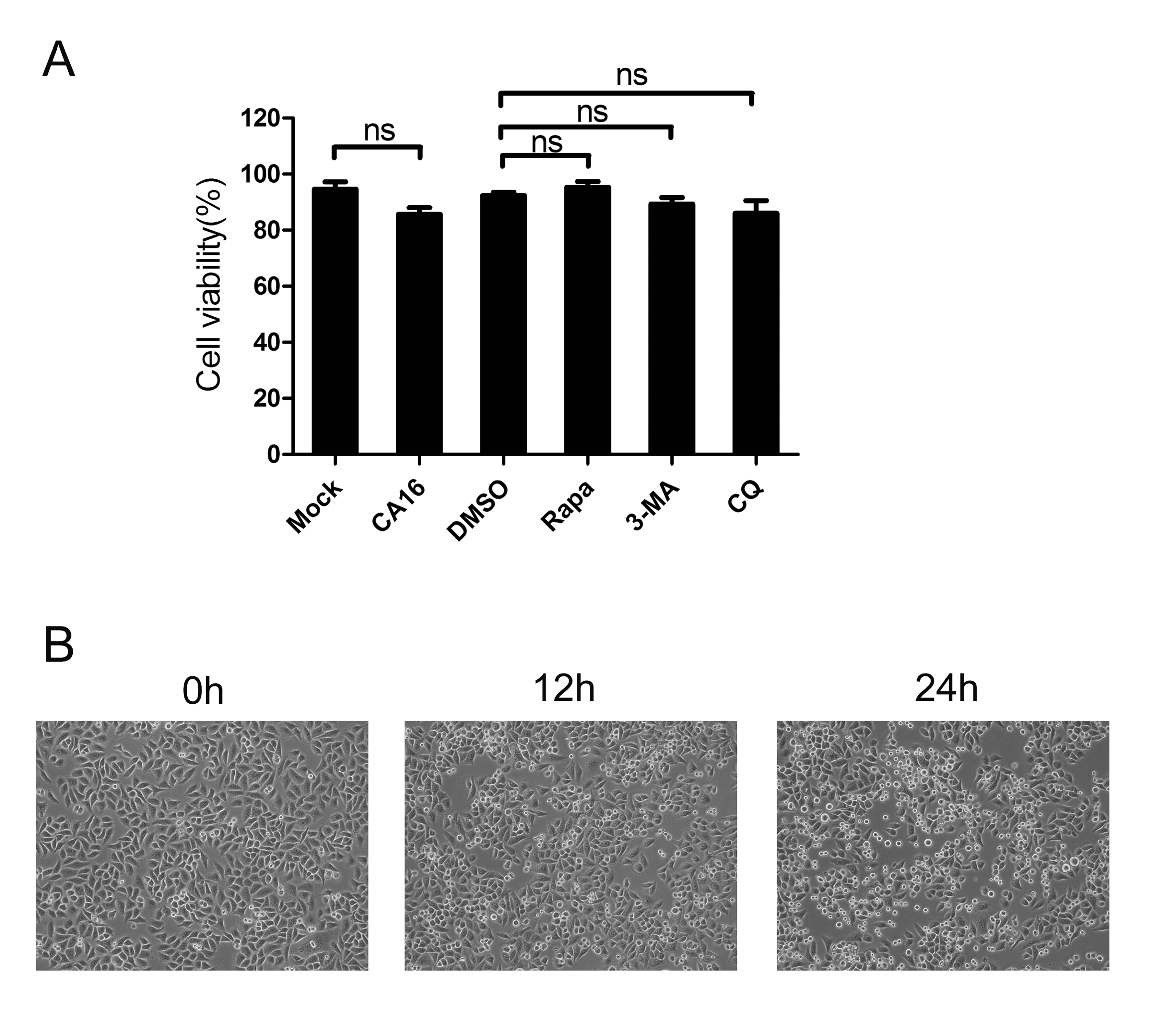

Supplement: S2 Fig — (A) Hela cells were infected with CA16 (MOI = 2) for 12h or were treated with optimal concentrations of rapamycin (Rapa, 100 nM), 3-methyladenine (3-MA, 5 mM) or chloroquine (CQ, 50 uM) for 24h and then cells viability was assessed by CCK8 analysis. All data are representative of at least three independent experiments, with each measurement performed in triplicate (mean ± SD of fold-change). *P< 0.05, **P< 0.01, ***P< 0.001. (B) The morphology of the infected Hela cells was investigated by microscopy at the indicated time points. Cells were infected with CA16 or not at an MOI of 0.1 and after 1 h of virus absorption at 37°C, the cells were further cultured in maintain medium. Cytopathic effect were observed at the indicated time points. (TIF) [file pone.0122109.s002.tif]

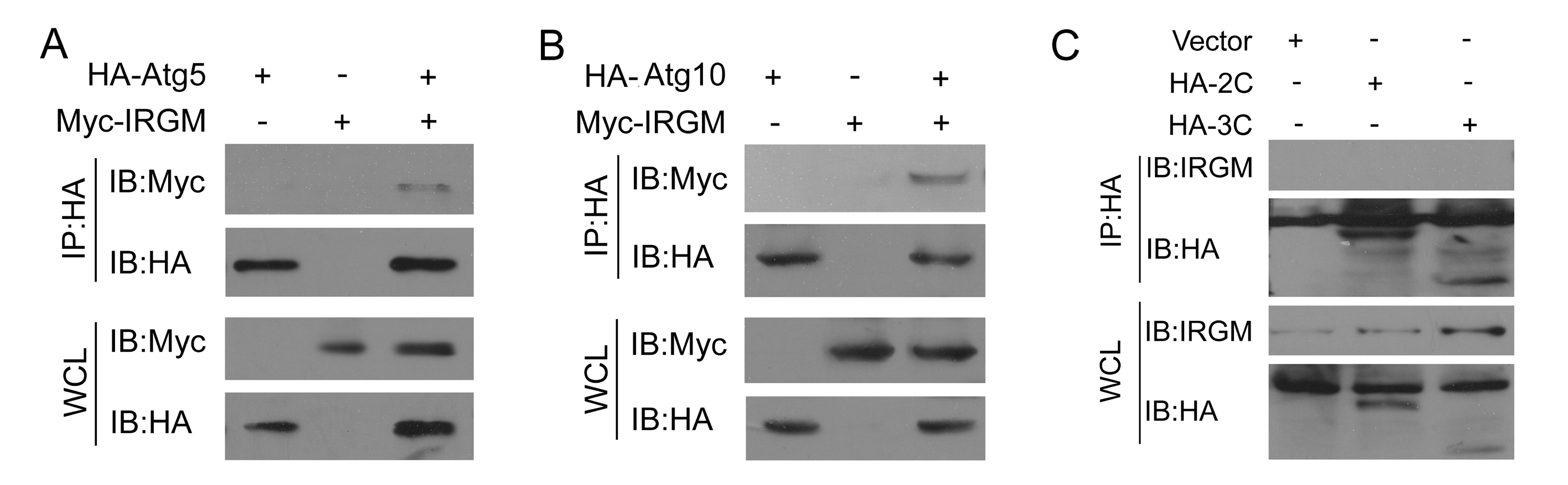

Supplement: S3 Fig — HeLa cells were co-transfected with HA-Atg5 (A) or HA-Atg10 (B) and Myc-IRGM for 24 h, followed by CA16 infection (MOI = 2) for 12h. Cell lysates were subjected to immunoprecipitation using anti-HA antibody followed by WB analysis with anti-HA and anti-Myc antibodies. (C) Hela cells were transfected with a vector, HA-2C or HA-3C constructs. Whole-cell lysates (WCL) were subjected to IP with anti-HA antibody, followed by WB with IRGM and HA antibodies. (TIF) [file pone.0122109.s003.tif]
