# Supplementary material for: Coxsackievirus A16 Elicits Incomplete Autophagy Involving the mTOR and ERK Pathways
Source: PLoS One. 2015 Apr 8;10(4):e0122109. doi: 10.1371/journal.pone.0122109 (PMC4390341; doi:10.1371/journal.pone.0122109)
Supplement: S1 Table — Bold And Italics, restriction endonuclease cutting sites. (DOC) [file pone.0122109.s004.doc]

**SUPPLEMENTAL FIGURE LEGENDS**

**Table 1 Primers used for the construction of various plasmids and qRT-PCR.**

| Primer name | Sequence(5’-3’) | purpose |
| --- | --- | --- |
| F-2A | ***GTCGAC***AGGAGAGTTTGGACAGCA | Cloning CA16-2A sequence for pCMV-HA-2A |
| R-2A | TT***GCGGCCGC***TTGCTCCATTGCTT |
| F-2B | C***GTCGAC***GGGAGTGTCTGATTATATCAA | Cloning CA16-2B sequence for pCMV-HA-2B |
| R-2B | GG***GGTACC***CTGTTTTTGCACTATGG |
| F-2C | GC***GTCGAC***TAGCGCTTCATGGCTA | Cloning CA16-2C sequence for pCMV-HA-2C |
| R-2C | GG***GGTACC***TTATTGGAAGAGGGCTTCTAT |
| F-3AB | C***GTCGAC***GGGACCCCCTAAGTTCAAG | Cloning CA16-3AB sequence for pCMV-HA-3AB |
| R-3AB | CG***GGTACC***TTGGACTGTGGCTGTCCTTA |
| F-3C | GA***GTCGAC***GGGACCGAGCTTAGACTTT | Cloning CA16-3C sequence for pCMV-HA-3C |
| R-3C | GG***GGTACC***TTATTGTTCACTGGCAA |
| F-3D | A***GTCGAC***AGGAGTGATCCAATGGATG | Cloning CA16-3D sequence for pCMV-HA-3D |
| R-3D | ATTT***GCGGCCGC***AAATAACTCGAGCC |
| F-IRGM | GC***GTCGAC***TATGGAAGCCATGAATGTT | Cloning IRGM sequence for pCMV-HA-IRGM |
| R-IRGM | G***GGTACC***TTAGTATTCACATACCCGC |
| F-LC3 | A***GAATTC***ATGCCGTCGGAGAAGACCT | Cloning LC3B sequence for pEGFP-N1-IRGM |
| R-LC3 | CG***GGATCC***CGCACTGACAATTTC |
| F-shAtg5 | ACAAGUUGGAAUUCGUCCAAA | 21-mer targets oligos for cloning into pLKO.1-TRC（Addgene） |
| R-shAtg5 | UUUGGACGAAUUCCAACUUGU |
| F-shBeclin1 | CAGUUUGGCACAAUCAAUAUU | 21-mer targets oligos for cloning into pLKO.1-TRC（Addgene） |
| R-shBeclin1 | AAUAUUGAUUGUGCCAAACUG |
| F-IRGM-P | AA***GGTACC***CGATGGCCCCTGGCAGTGA | Cloning IRGM promoter sequence for pIRGM-luc |
| R-IRGM-P | GA***AGATCT***CCACTGCTGGTTCGGGCA |
| F-IRGM-Q | CATTGTCTGGACCAAGCTAGAC | For real-time polymerase chain reaction (qPCR) |
| R-IRGM-Q | GTATTCACATACCCGCTCCTTC |
| F-GAPDH-Q | AATGGAAATCCCATCACCATCT | For real-time polymerase chain reaction (qPCR) |
| R-GAPDH-Q | CGCCCCACTTGATTTTGG |
| F-Atg5 | TC***GTCGAC***TATGACAGATGACAAAGA | Cloning Atg5 sequence for pCMV-HA-Atg5 |
| R-Atg5 | CG***GGTACC***TCAATCTGTTGGCTGTGG |
| F-Atg10 | GC***GTCGAC***TATGGAAGAAGATGAG | Cloning Atg10 sequence for pCMV-HA-Atg10 |
| R-Atg10 | GG***GGTACC***TTAAGGGACATTTCGTTC |
| F-IRGM-myc | GC***GTCGAC***TATGGAAGCCATGAATGTT | Cloning IRGM sequence for pCMV-myc-IRGM |
| R-IRGM-myc | G***GGTACC***TTAGTATTCACATACCCGC |

Bold And Italics, restriction endonuclease cutting sites.
